# Clinical and molecular findings in three Lebanese families with Bietti crystalline dystrophy: Report on a novel mutation

**Published:** 2012-05-05

**Authors:** Nour Maya N. Haddad, Naji Waked, Riad Bejjani, Ziad Khoueir, Eliane Chouery, Sandra Corbani, André Mégarbané

**Affiliations:** 1 Service d’Ophtalmologie, Hôtel-Dieu de France Hospital, Beirut, Lebanon; 2 Unité de Génétique Médicale. Faculté de Médecine, Université Saint-Joseph, Beirut, Lebanon; 3 Fondation ophtalmologique Adolphe de Rothschild, Paris, France

## Abstract

**Purpose:**

Bietti crystalline dystrophy (BCD) is a rare autosomal recessive disorder caused by mutation of the cytochrome P450, family 4, subfamily V, polypeptide 2 (*CYP4V2*) gene and characterized by retinal pigmentary abnormalities and scattered deposits of crystals in the retina and the marginal cornea. The aim of this study was to investigate the spectrum of mutations in *CYP4V2* in Lebanese families, and to characterize the phenotype of patients affected with BCD.

**Methods:**

Nine patients from three unrelated Lebanese families were clinically and molecularly investigated. Detailed characterization of the patients’ phenotype was performed with comprehensive ophthalmic examination, color vision study, fundus photography, visual field testing, retinal fluorescein angiography, electroretinography, and electrooculography. One family was followed for 12 years. The 11 exons of the *CYP4V2* gene were sequenced.

**Results:**

Symptoms consisting of night blindness, loss of paracentral visual field, and disturbed color vision were apparent during the third decade of life. Ophthalmoscopy revealed posterior pole crystalline deposits and areas of retinal pigment epithelium atrophy. Fluorescein angiography disclosed geographic areas of the pigment epithelium layer and choriocapillaris atrophy in the posterior pole and fundus periphery. The most striking findings were those of normal electroretinographic responses in some patients and clinical heterogeneity. Two mutations in *CYP4V2* were found: p.I111T (c.332T>C) in exon 3 in two families and the novel p.V458M (c.1372G>A) mutation in exon 9 in one family.

**Conclusions:**

These patients are affected with Bietti crystalline dystrophy without corneal involvement. Variation in disease severity and electroretinographic responses suggests that environmental or additional genetic factors influence the course of the retinal disease. The *CYP4V2* p.I111T (c.332T>C) mutant allele may be especially prevalent among patients with BCD in Lebanon, resulting from a single founder.

## Introduction

Bietti crystalline corneoretinal dystrophy (BCD; OMIM 210370) is a retinal degeneration first described by Bietti in 1937 [1,2]. BCD is characterized by multiple glistening intraretinal crystals scattered over the fundus, a degeneration of the retina, and sclerosis of the choroidal vessels, ultimately resulting in progressive night blindness and constriction of the visual field. Two types of BCD have been described: one with corneal crystalline deposits (one quarter to one third of patients with BCD) and a second type with no corneal involvement and no limbal crystalline deposits, called Bietti crystalline fundus dystrophy (BCD) or crystalline retinopathy [1]. The mode of inheritance is autosomal recessive, and mutations of the cytochrome P450, family 4, subfamily V, polypeptide 2 (*CYP4V2*) gene, a novel family member of the cytochrome P450 genes widely expressed in the human heart, brain, placenta, lung, liver, and most abundantly in the retina and the retinal pigment epithelium, have been reported [1-9].

BCD is a global disease that appears to be more common in individuals of Asian descent [5,10]. In the Middle East, a few cases have been reported [11] but none molecularly investigated.

The purpose of this paper is to report for the first time on the clinical and molecular analysis in three Lebanese families with BCD, discuss the genotype-phenotype correlations as well as expose a 12-year follow-up in one family in which a novel mutation was identified.

## Methods

This study was granted approval by the Hotel-Dieu de France of Beirut Committee on Clinical Investigation and conformed to the tenets of the Declaration of Helsinki.

### Clinical investigations

**Figure 1:**
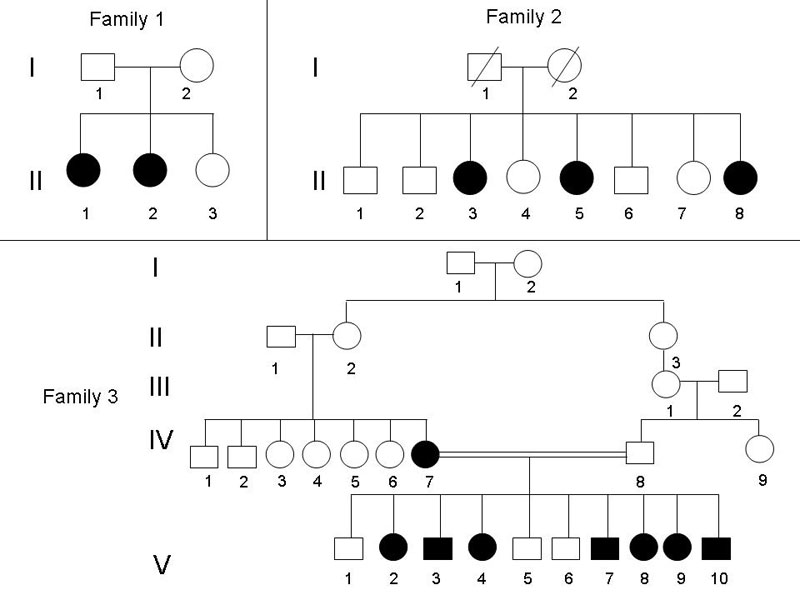
Pedigrees of the three Lebanese families with Bietti crystalline dystrophy. Affected individuals are indicated by filled symbols. DNA was available for all members of family 1, for individuals II-3 and II-5 of family 2, and for individuals IV-7, V-2, V-3, and V-9 of family 3.

Otherwise good health except eye findings, one Maronite and 2 Shiite unrelated Lebanese families with clinical criteria of BCD syndrome were investigated (Figure 1). Nine affected individuals were examined extensively, including a comprehensive ophthalmic examination, color vision study, fundus photos, automated and Goldmann perimetry, routine blood tests, and molecular analysis.

### Molecular studies

After informed consent was obtained from all the families, EDTA blood samples were collected, and DNA extracted on the spot for genetic studies. Eleven DNA samples were collected for this study from members of the three families (Figure 1): nine affected individuals and two parents (family 1:I-1 and I-2). DNA was extracted from lymphocytes with standard salt-precipitation method.

### Polymerase chain reaction amplification

The *CYP4V2* gene is made up of 11 exons. Intronic primers were designed using the Primer 3 software (Table 1). DNA sequences were obtained from the Human Genome Browser (UCSC database access number NM_207352) by comparing genomic DNA with cDNA sequences.

**Table 1:** PCR primers for the 11 exons of the *CYP4V2* gene.

**Primers**	**Forward**	**Reverse**
Exon 1	TTTTCCGGTCTTTCGCTTC	AAATGCTCACTTTGGGATGG
Exon 2	CAGGCAGTTCACACATGCTC	TGGAAAGCATTAAATTGCACC
Exon 3	AATTACAGGAAGGTTGTTTGATG	TTCTTGAAATAACAAGTTGCACG
Exon 4	CGTTTTGGATGTTACTTTTCTCTTTC	TTCCTGTTTGGGCCATTTTC
Exon 5	ACGTCTTTAGTGTCTGCCGC	GATACAACGCAGAAATTGTTAGC
Exon 6	GACAATCATCGTCATTCCCAC	CATCGTGAATGCACTTAATACC
Exon 7	TCACAAGAGCCTATGTTGTCG	AAGAAGTTGAGCTGGTACTTAATCAG
Exon 8	TCACTCCTAATCATCGCAGC	GCCTTCCTGCTCATTACACTG
Exon 9–10	CACCTGTTCTTTTTAGATGTCTGC	TGTGAGAAACCCACCATCAA
Exon 11	TTCTCCTTCCACCTACTGCG	TTAGGTCTAGGGGATTCAAGC

### Fluorescent sequencing

All PCR-amplified fragments of the 11 exons of *CYP4V2* gene were fluorescently sequenced using an ABI 3130 automated sequencer (Applied Biosystems, Foster City, CA). Electropherograms were compared to reference sequences using the Sequencher software v4.2 (Gene Codes Corporation, Ann Arbor, MI) and ChromasPro v1.5 (Technelysium, Tewantin, Australia).

### Polymerase chain reaction-restriction fragment length polymorphism

Fifteen µl of the amplified product including a novel identified variant were digested with 7.5 units of the NlaIII restriction enzyme (New England Biolabs Inc., Ipswich, MA) overnight and electrophoresed on 2% agarose.

## Results

### Clinical findings

**Figure 2:**
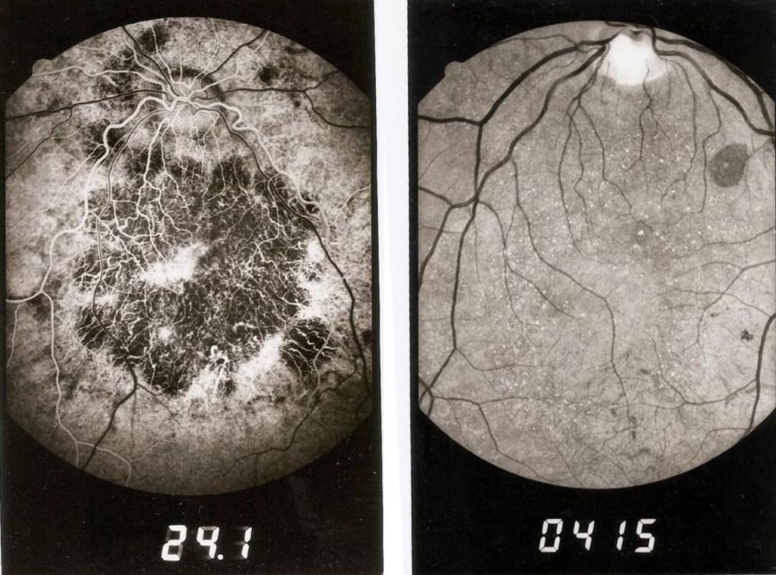
Numerous retinal crystals and chorioretinal atrophy are visible on the fundus photos of the patients with Bietti crystalline corneoretinal dystrophy.

Fifteen patients were examined in total. Nine patients were affected, and the remaining six asymptomatic individuals were examined in the genetic counseling setting. The clinical findings from the nine affected patients are summarized in Table 2. Eight were female. Two were asymptomatic, three had visual complaints related to nyctalopia, and four complained of decreased visual acuity (VA). Three of the patients had blindness, while in the others, visual acuity was preserved even in severe cases since the central vision had remained normal at first particularly for near, before decreasing gradually. Best-corrected visual acuity (BCVA) in these individuals ranged from light perception to Snellen 20/20. Color vision testing using Ishihara plates ranged between normal and severe color vision deficiency. Automated visual fields when performed gave results ranging between paracentral scotoma and severe uniform alteration. The slit lamp exam did not show any corneal deposits, and the anterior segments were within normal limits in all examined patients. Fundus photos showed chorioretinal atrophy in all affected patients and crystalline deposits in some cases (Figure 2). When performed, electroretinography (ERG) remained within normal limits, and electrooculography (EOG) showed an additional decrease in the Arden ratio that was either moderately or severely affected.

**Table 2:** The clinical data of the 9 patients with Bietti crystalline dystrophy.

**Patients/AGE**	**Sex**	**Symptoms**	**BCVA**	**VF**	**Anterior segment**	**Fundus**	**ERG**
F1:II-1/ 31yrs	F	nyctalopia	20/40 OD 20/25 OS	paracentral infero-nasal scotoma OD and a moderate cæcal scotoma OS	WNL	crystalline deposits, choriocappilaris and RPE atrophy	WNL
F1:II-2/ 29yrs	F	nyctalopia	20/25 OU	paracentral scotoma OU	WNL	crystalline deposits, choriocappilaris and RPE atrophy	WNL
F2:II- 3/34yrs	F	asymptomatic	20/20 OU	NA	WNL	crystalline deposits, choriocappilaris and RPE atrophy	NA
F2:II-5/30yrs	F	blurry vision	20/200 OD 20/30 OS	paracentral scotoma OU	WNL	crystalline deposits, choriocappilaris and RPE atrophy	NA
F2:II-8/ 22yrs	F	asymptomatic	20/20 OU	NA	WNL	crystalline deposits, choriocappilaris and RPE atrophy	NA
F3:IV-7/ 65yrs	F	blindness	CF 50cm OU	blind	WNL	Chorioretinal atrophy	NA
F3:V-2/ 39yrs	F	blindness	LP OU	blind	WNL	Severe Chorioretinal atrophy and RD	NA
F3:V-3/ 37yrs	M	blindness	20/400 OD CF 50cm OS	blind	WNL	Chorioretinal atrophy	NA
F3:V-8/ 26yrs	F	nyctalopia	20/25 OD 20/30 OS	paracentral scotoma OU	WNL	crystalline deposits, choriocappilaris and RPE atrophy	NA

F: Family; C:case; BCVA: Best Corrected Visual Acuity; VF: Visual Field; ERG: Electroretinogram; OD: Right Eye; OS: Left eye; OU: both eyes; WNL: Within Normal Limits; NA: not recorded.

Two patients from family 1 (Figure 1) were followed up for 12 years. Patient 1:II-1 initially had a BCVA of 20/40 OD and 20/25 OS in 1991. BCVA remained stable for approximately five years and began to drop progressively to reach 20/50 OU in 1999. The last follow-up examination at year 12 in 2003 showed a dramatic decrease in BCVA that became 20/120 OU with subtle changes in ocular fundoscopy where more chorioretinal atrophy and fewer crystalline deposits were noted. The cornea and anterior chamber remained normal during the entire follow-up, but small equatorial lens opacities were found in both eyes at year 1997 and remained stable afterwards. ERG was performed in 1996; results were within normal limits except for a slightly diminished maximal response whereas the EOG results showed a decrease in the Arden ratio. Patient 1:II-2 had an initial BCVA of 20/25 OU in 1991. BCVA OD remained stable during the first seven years and then dramatically dropped to 20/120 at the end of 1999 when the ocular fundus began to show more macular chorioretinal atrophy with fewer crystalline deposits. BCVA OS remained stable during the entire follow-up period reaching 20/30 in 2003. Small blue equatorial lens opacities were found in both eyes in 1999 and remained unchanged afterwards. ERG findings in 1996 were strictly normal, but the EOG showed a decrease in the Arden ratio that was more important in the left eye.

### Molecular analysis

**Figure 3:**
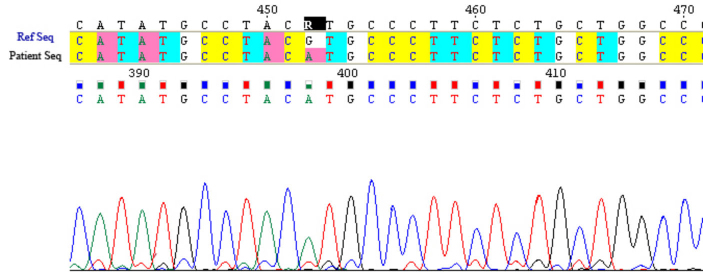
Electropherogram of the identified mutation: c.1372G>A in the *CYP4V2* gene. “Patient Seq” represents the affected individual sequence, homozygous for the mutation compared to the reference sequence “Ref Seq.”

The exploration of the entire coding sequence of *CYP4V2* in our series allowed identification of the causative mutation in the three families. In family 1, a novel homozygous variation, p.V458M (c.1372G>A), was found in exon 9 in the affected individuals, and at the heterozygous state in their non-affected parents (Figure 3). This mutation was confirmed with polymerase chain reaction-restriction fragment length polymorphism. The 550 bp amplified product was digested by NlaIII producing two fragments of 495 and 93 bp in the affected patients with an undigested product of 545 bp in the heterozygous non-affected individuals.

**Figure 4:**
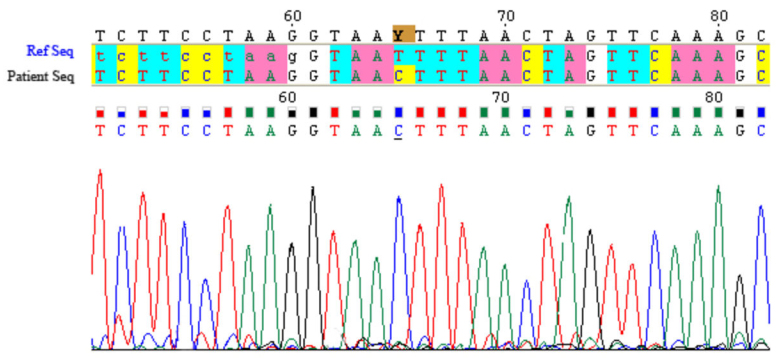
Electropherogram of the identified mutation: c.332T>C in the *CYP4V2* gene. “Patient Seq” represents the affected individual sequence, homozygous for the mutation compared to the reference sequence “Ref Seq.”

In families 2 and 3, a missense mutation p.I111T (c.332T>C) was found in exon 3 at the homozygous state in the affected individuals (Figure 4). Random DNA samples from 400 Lebanese chromosomes were subsequently tested for these mutations with fluorescent sequencing, and the variations were absent in all chromosomes.

## Discussion

Here, we report three Lebanese families with Bietti crystalline fundus dystrophy (BCD) or crystalline retinopathy. BCD affects both sexes, and the onset of features usually occurs during the third decade of life, as was the case with our patients. Night blindness and symptoms related to loss of the peripheral visual field are prominent. Color vision may also be disturbed. Even in the asymptomatic stage, retinal crystalline deposits were already readily visible [12]. BCD has been described as a progressive disease, with a variable rate of progression as illustrated in the present families.

Functional tests can vary widely in the presence of identical morphologic changes in the fundus. In our cases, fluorescein angiography (FA) performed in the two sisters from the first family confirmed the diagnosis by revealing geographic areas of the retinal pigment epithelium and choriocapillaris atrophy involving the macular, perimacular, and peripapillary regions in both eyes (Figure 2). ERG within the normal range, such as in the two affected sisters in F1, has rarely been reported [13]. The most striking findings were those of the first family with two sisters having decreased visual acuity and progressive disease while maintaining a normal ERG. Usually, ERG is borderline to moderately abnormal in the localized form, and severely abnormal in the diffuse form. Abnormalities involve the photopic and scotopic systems. Researchers have suggested that cones might be less involved than rods in BCD, and that progression of BCD is consistent with a rod-cone dystrophy pattern [14].

**Figure 5:**
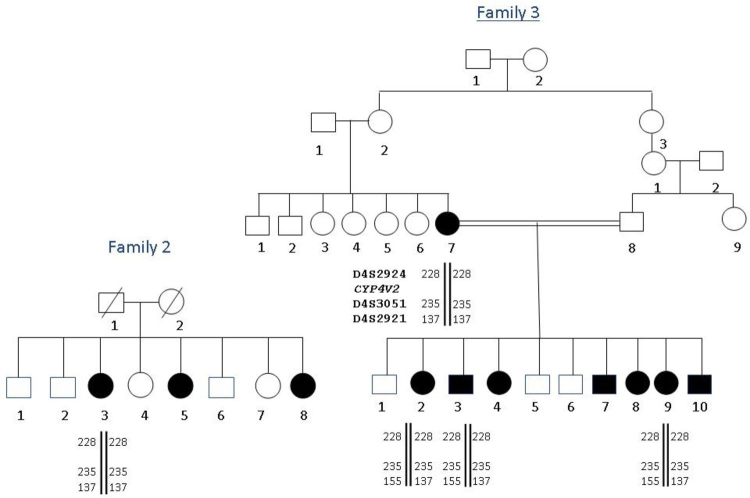
Haplotypes of families 2 and 3 using D4S2924, D4S3051, and D4S2921 STR markers. Alleles are given in bp.

The analysis of the *CYP4V2* gene showed an abnormality in the three families. In family 1, a novel mutation was found: p.V458M (c.1372G>A). This variation was predicted as damaging to the protein function according to SIFT (Sorting Intolerant From Tolerant) software. A 12-year follow-up showed a mild exaggeration of symptoms, with progressive and moderate visual acuity reduction with the surprising findings of normal ERGs. This suggests the existence of less severe forms of BCD related to relatively mild *CYP4V2* mutations. In families 2 and 3, a previously reported mutation was found, the p.I111T (c.332T>C) [10]. Both families denied any relation with each other. However, as the families share the same haplotype (Figure 5), we can postulate that this mutation derived from an ancestral mutation that was geographically restricted. Clinical comparison of the families did not show any phenotype-genotype correlation. Indeed, at the age of 39 and 37, patients V-2 and V-4 of family 3, respectively, presented a severe clinical phenotype; whereas patients II-3 and II-5 of family 2 aged 34 and 30, respectively, presented a less severe disease suggesting the possible involvement of genetic, epigenetic, or even environmental factors in the pathogenesis of BCD. In terms of environmental factors, lipid metabolism, and therefore diet, may play a role in the disease. Indeed, researchers have shown that *CYP4V2* may play a role in fatty acid and steroid metabolism, which might be consistent with biochemical studies of patients with BCD [6,15]. Abnormal choroidal fibroblast inclusions are similar to those found in circulating lymphocytes, keratocytes, and conjunctival and skin fibroblasts [3]. Cultured lymphocytes from patients with BCD lack two fatty acid-binding proteins of 32 and 45 kDa, in comparison to age-matched controls [16].

In conclusion, this study further confirms the role of *CYP4V2* in the pathogenesis of BCD. We report a new mutation that is associated with a better prognosis and normal ERG. Longitudinal studies on patients with BCD may reveal further differences in the clinical phenotype and severity of the disease according to their genotype.
